# Rhino-orbital mucormycosis following severe COVID-19 infection

**Published:** 2021-07-20

**Authors:** Tarjani Vivek Dave, Savitri Sharma

**Affiliations:** 1 Associate Ophthalmologist, Ophthalmic Plastic Surgery Service: LV Prasad Eye Institute, Hyderabad, India.; 2 Microbiologist, Jhaveri Microbiology Centre: LV Prasad Eye Institute, Hyderabad, India.

**Figure:**
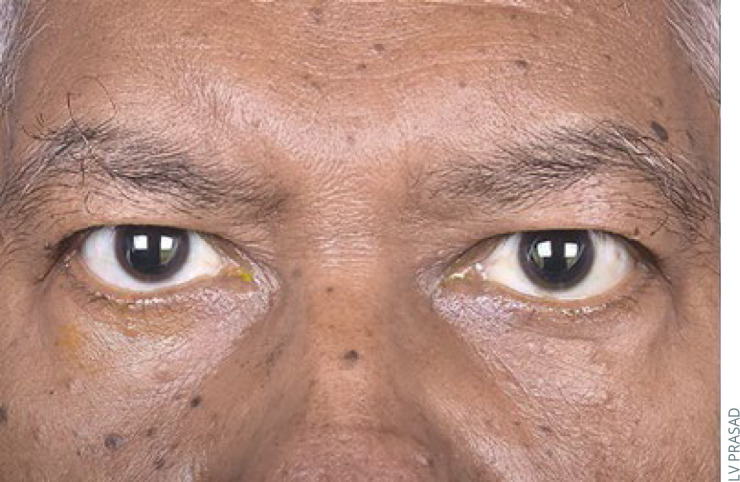
Figure 1

**Figure:**
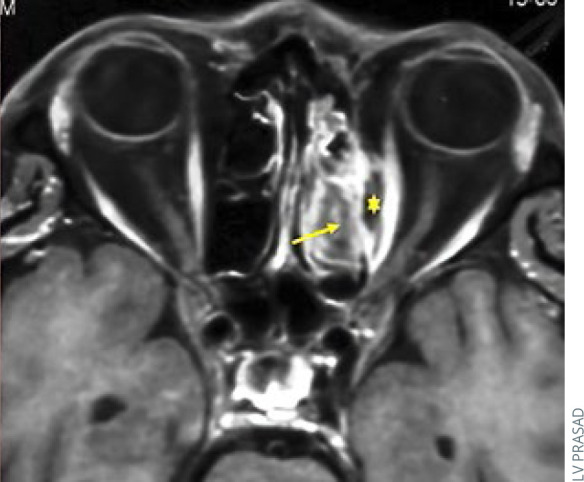
Figure 2

A 49-year-old man presented with complaints of left-sided nasal stuffiness, peri-orbital pain and double vision. He did not have any other systemic complaints and was non-pyretic. He was known to have hypertension and diabetes, for which he had been taking oral medication for nine years, controlled based on random blood sugar testing at home with a glucometer (range of 140 to 200 mg/dl).

The man had recovered from COVID-19 just three weeks earlier; he had developed pulmonary involvement due to COVID-19. His inflammatory markers were also raised. He was treated with intravenous methylprednisolone 40 mg daily for 5 days, followed by oral prednisolone in tapering doses for 15 days, for moderate to severe COVID-19 disease. During this phase of treatment with IV steroids, the patient’s blood sugar values were uncontrolled and he was prescribed insulin to improve blood sugar control. One week after completing the course of steroids, he presented to the ophthalmology clinic complaining of double vision.

On examination, the patient had mild proptosis of the left eye (Figure 1) and a diagnostic nasal endoscopy was suggestive of an eschar in the nostril over the middle turbinate. A contrast-enhanced MRI of the paranasal sinuses and the brain (Figure 2) demonstrated left ethmoid sinusitis (**arrow**) with a medial orbital abscess that was not taking up contrast (*). A diagnosis of post COVID-19 invasive fungal sinusitis with orbital involvement, presumed rhino-orbital mucormycosis, was made.

**Question 1**

**What are the risk factors for invasive fungal sinusitis in this patient?**

- Recently recovered from COVID-19
- Known hypertensive
- Treated with IV steroids for COVID-19
- Options a and c
- All of the above

**Question 2**

**Which tests and procedures would you perform next?**

- Complete ophthalmic examination
- Fasting and postprandial blood sugar with HbA1C
- Endoscopic endonasal sinus debridement with medial wall decompression and drainage of the medial orbital abscess
- Send pus from the involved areas for microbiology and tissue specimens for histology
- All of the above

**Question 3**

**MRI imaging (Figure 2) is suggestive of ethmoid sinus haziness with a focal medial orbital abscess without contrast uptake. What would you do next?**

- Sinus debridement
- Sinus and orbital debridement
- Sinus and orbital debridement with local transcutaneous retrobulbar amphotericin B
- Sinus and orbital debridement with local transcutaneous retrobulbar amphotericin B with intravenous liposomal amphotericin B (dose of 3-5 mg/kg body weight)

## ANSWERS

(d) Uncontrolled blood sugar level is one of the most important risk factors for mucormycosis. Infection with the SARS-CoV2 virus leads to uncontrolled blood sugar levels, especially in those who already have diabetes. Use of intravenous steroids will also lead to increased blood sugar levels, predisposing to fungal infection. Hypertension is not a known risk factor for mucormycosis.
(e) All of the above-mentioned tests and procedures are required to make a complete diagnosis of rhino-orbital mucormycosis
(d) This patient has a rhino-orbital presentation of mucormycosis. Intravenous liposomal amphotericin B is the drug of choice for all patients with mucormycosis and can be initiated where there is a strong clinical suspicion, even before microbiology or histology results become available. Similarly, for all suspected cases, necrotic sinus tissue is debrided to reduce the disease load. This is done for two reasons: (i) the disease can spread to the brain quickly, and brain involvement is associated with high mortality (80%) and (ii) since the orbital involvement lacks contrast uptake, it suggests that there is angioinvasion in that area, requiring debridement and local retrobulbar amphotericin B injections.

